# Thermodynamics Study of Solvent Adsorption on Octadecyl-Modified Silica

**DOI:** 10.1007/s10337-014-2788-4

**Published:** 2014-10-30

**Authors:** Szymon Bocian, Jan Soukup, Pavel Jandera, Bogusław Buszewski

**Affiliations:** 1 Department of Environmental Chemistry and Bioanalytics, Faculty of Chemistry, Nicolaus Copernicus University, Gagarin 7 St, 87-100 Torun, Poland; 2 Department of Analytical Chemistry, Faculty of Chemical Technology, University of Pardubice, Pardubice, Czech Republic

**Keywords:** Column liquid chromatography, Solvent adsorption, Temperature influence, Enthalpy, Entropy

## Abstract

Elution and solvation processes in liquid chromatography may be controlled by temperature changes. In the case of solvent adsorption, the temperature influences the amount of adsorbed solvent as well as the enthalpy and entropy of the solvation process. In this work, the thermodynamic parameters of organic solvents used as organic modifiers in the reversed-phase high-performance liquid chromatography elution process were determined. The changes of enthalpy and entropy in a series of chemically bonded stationary phases were measured to determine the effects of the temperature and surface coverage density of octadecyl ligands on the thermodynamic parameters of the solvation. For both the enthalpy and entropy a parabolic trend was observed with the minimum for medium surface coverage. The correlation of solvent adsorption values with the enthalpy of solvation was also investigated. The highest influence of the temperature on solvation process was observed for stationary phases with high surface coverage.

## Introduction

When a mixed mobile phase is in contact with the hydrophobic surface of the stationary phase, less-polar solvent molecules can preferentially adsorb on the surface. The composition of the mobile phase at the surface is different from its bulk composition [1–3]. In reversed-phase liquid chromatography system, the acting stationary phase is a combination of three components: bonded ligands, residual silanols and adsorbed molecules of both organic solvent and water [4–6].

Many studies were conducted to explore the retention mechanism in reversed-phase liquid chromatography (RPLC) and two main theories were taken into account [7]. Retention mechanism in RPLC has been described by solvophobic theory introduced by Horvath and co-workers [8, 9] suggesting that the retention is especially influenced by hydrophobic interactions between the mobile phase and solutes and is considered to occur through non-localized adsorption rather than partitioning. The driving force for retention as described by solvophobic theory is the free energy change associated with the two-step solute-transfer mechanism including creation of a solute-sized cavity in the mobile phase and transfer of the solute to or from this cavity. The free energy change can be quantified by measuring the partition coefficients of non-polar molecules between water and non-polar solvents.

In a more recent partitioning model, Dill [10] has proposed a three-step process involving creation of a solute-sized cavity in the stationary phase, transfer of the solute from the mobile phase to the stationary phase, and closing of the solute-sized cavity in the mobile phase. The solute is approximated to be fully embedded in the stationary-phase chains rather than adsorbed on the surface and this model also supposes that the retention process is especially driven by two forces. One is the difference in the contact-free energy of the solute in the mobile phase and the stationary phase. The second driving force for retention is related to the partial ordering of the non-polar groups bonded onto the surface of stationary phases, leading to entropic ejection of the solute from the stationary phase at sufficiently high bonding density. At low bonding densities, the partitioning of solutes should increase linearly up to a critical bonding density of about 2.7 μmol m^−2^ suggested by Cole and Dorsey [11].

It is commonly believed that retention in the RPLC is controlled by the distribution of solute between the bulk mobile phase and the stationary phase and interactions of ligands bonded on stationary-phase surface with solute are primarily influenced by hydrophobic effect proposed. The concentration of mobile phase at the surface of stationary phase varies from its bulk concentration [3]. The residual silanols may also play very important role in adsorption of solute [6]. Indeed the retention of solute is governed by partitioning between the layer of bonded non-polar groups onto the stationary phase or by adsorption or their combination [12].

The partition coefficients can be transformed to free energy (Δ*G*) of transfer involving enthalpic (Δ*H*) and entropic (Δ*S*) components.

1 $$ \Delta G\,{ = }\,\Delta H{-}T\Delta S $$

These components may be experimentally determined by calorimetry which has been reported frequently in the literature [13]. The relationship between the free energy, retention factor, *k*, and thermodynamic temperature, *T* (in Kelvin) [14], is described according to the following expression (van’t Hoff equation) [9]:

2 $$ \ln k_{i} = \ln K + \ln \frac{{V_{\text{S}} }}{{V_{\text{M}} }} = \frac{{ - \varDelta G^{0} }}{\text{RT}} + \ln \frac{{V_{\text{S}} }}{{V_{\text{M}} }} = \frac{{\varDelta S^{0 } }}{R} + \ln \frac{{V_{\text{S}} }}{{V_{\text{M}} }} - \frac{\varDelta H^0 }{\text{RT}} = A_{\text{i}} + \left( {\frac{{B_{\text{i}} }}{T}} \right) $$

In this case, the ln *k* versus *T* ^−*1*^ plots should embody linearity [15–17]. The parameter *B*_*i*_ involves the standard partial molar enthalpy of transfer of the solute *i* from the mobile phase to the stationary phase, −Δ*H* ^0^. The parameter *A*_*i*_ is proportional to the standard partial molar entropy of the transfer of the solute from the mobile phase to the stationary phase, Δ*S* ^0^, and includes the phase ratio (the ratio of the volumes of the stationary, *V*_S_, and of the mobile, *V*_M_) in the chromatographic system as well [18]. The *V*_M_ is the column hold-up volume essential for the determination of the retention factor, *k*. *R* is the gas constant and *T* is the thermodynamic temperature (in Kelvins) [19–21]. By plotting ln *k* versus *T* ^−*1*^ over a sufficiently broad temperature range, it may be calculated the enthalpic and the entropic contributions to retention and selectivity, −Δ*H* ^0^ from the slope and Δ*S* ^0^ from the intercept of the plot. van’t Hoff plots can provide the information on whether or not the retention mechanisms change over the studied temperature range [22, 23]. It has been acknowledged that phase transition phenomenon may cause some small deviations from linearity [24, 25]. That is the consequence of a change in the molecular structure of the stationary phase which appears in the 20–50 °C range for C18 silica-based stationary phases. This phenomenon is quite independent of the nature of both solute and eluent. However, it was shown that these deviations were relevant only for stationary phases with high bonding density (bonding densities higher than 4.0 μmol m^−2^) [11, 25].

Non-linear plots of ln *k* versus 1/*T* have been observed more rarely [26, 27]. van’t Hoff equation describing curvilinear dependency of ln *k* on 1/*T* can be obtained for the best fit of a curved van’t Hoff plot by the partial derivative of ln *k’* with respect to 1/*T* yielding a second equation (Eq. 3) [28].

3 $$ \ln k = b_{0} + \frac{{b_{1} }}{T} + \frac{{b_{2} }}{{T^{2} }} + \ln \varPhi $$

In this case, both Δ*H* ^0^ and Δ*S* ^0^ vary with *T* according to the following equations:

4 $$ \varDelta H^0 = - R\left( {b_{1} + \frac{{2b_{2} }}{T}} \right) $$

5 $$ \varDelta S^0 = R\left( {b_{0} - \frac{{b_{2} }}{{T^{2} }}} \right) $$

6 $$ \ln k = - \left( {\frac{\varDelta G}{\text{RT}}} \right) + \ln \left( {\frac{{V_{\text{S}} }}{{V_{\text{M}} }}} \right) $$

*b*_0_ *, b*_1_ and *b*_2_ are the parameters of Eq. 3 which can be determined from non-linear regression and consequently used for calculations of system enthalpy, Δ*H*, and entropy, Δ*S*. The classical linear van’t Hoff behavior is related to a change in the heat capacity of the system independent on *T* [29, 30] and the non-classical van’t Hoff behavior to a change in the heat capacity dependent on *T*.

Although RPLC was thoroughly investigated many times, only few works devoted the attention to effect of temperature on retention mechanism especially in low temperatures. These are not commonly used in ordinary high-performance liquid chromatography (HPLC) applications but temperature dependency of retention in broad range can provide description of the energetic principles of chromatographic process.

The goal of this study was to determine the thermodynamic parameter of organic solvents used as organic modifiers in reversed-phase high-performance liquid chromatography (RP HPLC) elution process. The changes of enthalpy and entropy on a series of chemically bonded stationary phases were measured to determine, how the temperature and coverage density influence the thermodynamic parameters. The correlation of solvent adsorption values with the enthalpy of solvation was also investigated.

## Experimental

### Instruments

The phases under study were packed into 125 mm × 4.6 mm. stainless steel columns. All columns were packed using a DT 122 packing pump (Haskel, Burbank, CA, USA) under the pressure of 40 MPa.

An HP Model 1050 (Agilent Technologies, Waldbronn, Germany) liquid chromatograph was equipped with four-channel gradient pump, an autosampler with a 100-μL loop, diode array UV–Vis detector and computer data acquisition station.

Temperature was controlled using liquid thermostat Julabo F12 (Julabo Labortechnik GmbH, Seelbach, Germany), with the temperature stability ±0.03 °C.

### Materials

The solid support of in-house made phases was Kromasil^®^ 100 (Akzo Nobel, Bohus, Sweden). Five chromatographic columns with different surface coverage densities were studied. All the columns were prepared from the same batch of silica particles. The silica was chemically modified with octadecyldimethylchlorosilane (Wacker GmbH, Munich, Germany). Morpholine was purchased from Sigma-Aldrich Chemie (Steinheim, Germany). The characteristics of these stationary phases are listed in Table 1. Columns were not end-capped.

**Table 1:** Coverage density of stationary-bonded phases

Phase code	Carbon load (*P*_c_) (%)	Hydrogen load (*P*_H_) (%)	Coverage density (*α*_RP_) (µmol m^−2^)
#1	2.42	0.93	0.33
#2	7.55	1.73	1.11
#3	10.88	2.24	1.68
#4	17.27	1.14	2.95
#5	18.70	3.33	3.27

Geometrical parameters of the packed columns and bare silica gel (pore volume, void volume, external porosity and phase ratio) were obtained using inverse size exclusion chromatography [31] and they are listed in Table 2. These data were used for calculation of the phase ratio.

**Table 2:** Geometric parameters of the columns measured by inverse size exclusion chromatography

Phase code	Column void volume (*V*_M_) (mL)	Interparticle volume (*V*_ex_) (mL)	Pore volume in the column (*V*_pore_) (mL)	External porosity (*ε*_e_)	Stationary-phase volume (*V*_s_) (mL)	Phase ratio
#1	1.711	0.878	0.833	0.42	0.365	0.213
#2	1.547	0.851	0.697	0.41	0.529	0.342
#3	1.437	0.820	0.618	0.39	0.639	0.445
#4	1.251	0.778	0.473	0.37	0.826	0.659
#5	1.247	0.801	0.446	0.39	0.829	0.665

All solvents were HPLC *“isocratic grade”*, purchased from J.T. Baker (Deventer, The Netherlands). Water was purified using Milli-Q system (Millipore, El Paso, TX, USA). All eluents were degassed in ultrasonic bath under vacuum.

### Measurements

Measurements were carried out with flow rate 1 ml/min at the temperature 295 K. For the temperature dependences, the measurements were carried out in 278, 288, 298, 308, 318, and 328 K (5, 15, 25, 35, 45, and 55 °C). The measurements of the solvent retention were repeated in triplicate and arithmetic means of the experimental retention times, *t*_R_, and the column hold-up time, *t*_M_, were used to calculate the retention factors, *k* = *(t*_R_−*t*_M_ *) t*_M_^−1^. Organic solvent was injected onto the plateau at given mobile-phase composition. Data were corrected according to the void volume of the apparatus. The highest relative error of retention time measurement was 0.90 %, whereas for most measurement it was lower than 0.25 %.

The thermodynamic void volume of the column (*V*_M_) for excess isotherms calculations was obtained by integrating the plot of the retention times of the perturbation peaks (*V*_R_) from 0 to 100 % of the organic modifier [32]:

6 $$ V_{\text{M}} = \frac{1}{{C_{{max} } }}\int\limits_{C = 0}^{{C = C_{{max} } }} {V_{\text{R}} \left( C \right){\text{d}}C} $$

where *C*_max_ is a maximum concentration of the organic modifier in the mobile phase.

When *V*_M_ is known, the excess amount of the adsorbed organic modifier per unit amount of stationary phase (*Γ*) can be calculated [33]:

7 $$ \varGamma (C) = \frac{1}{{S_{\text{BET}} }}\int\limits_{0}^{C} {\left( {V_{\text{R}} \left( C \right) - V_{\text{M}} } \right){\text{d}}C} $$

where *S*_BET_ is a total surface area of the stationary phase (m^2^).

The maximum concentration of the adsorbed solvent (*C*_ads_) can be found by extrapolating the slope of the excess isotherm in a linear region to the *y*-axis. In this work, *C*_ads_ was calculated as the intercept parameter of the straight line fitted to the linear region of the excess isotherm.

The result of non-linear fitting of van’t Hoff plot, according to Eqs. 3 is listed in Table 3. Enthalpy (Δ*H*), entropy (Δ*S*) and Gibbs free energy (Δ*G*) were calculated based on Eqs. 4, 5 and 6. The volumes for phase ratio calculations were measured using ISEC and they are listed in Table 2.

**Table 3:** Parameters *b*_0_, *b*_1_, and *b*_2_ of Eq. (3) fitted to van’t Hoff plot and determination coefficients, *R* ^2^

*α*_RP_^*I*^ (μmol m^−2^)	MeOH				PrOH			
	*b*_0_	*b*_1_	*b*_2_	*R* ^2^	*b*_0_	*b*_1_	*b*_2_	*R* ^2^
3.27	−40.81	22,813	−3,215,289	0.9929	−18.52	11,409	−1,582,457	0.9689
2.95	−13.97	7,718	−1,086,207	0.9982	−10.04	6,898	−982,707	0.9905
1.68	−2.11	501.9	−34,058	0.9568	−4.28	3,523.6	−518,811	0.8930
1.11	−2.13	1,095	−142,960	0.9735	−5.63	4,097	−607,148	0.9512
0.33	−6.06	2,626	−344,405	0.9962	−7.13	3,963	−535,798	0.9926

*α*_RP_^*I*^ (μmol m^−2^)	EtOH				ACN			
	*b*_0_	*b*_1_	*b*_2_	*R* ^2^	*b*_0_	*b*_1_	*b*_2_	*R* ^2^
3.27	−30.17	17,415	−2,452,454	0.9876	−31.00	17,645	−2,452,898	0.9949
2.95	−12.20	7,376	−1,044,127	0.9944	−11.71	6,815	−921,506	0.9991
1.68	1.86	1,039.1	−131,836	0.9899	−3.85	2,014.9	−241,089	0.9962
1.11	−2.00	1,462	−206,583	0.9273	−2.06	1,237	−127,311	0.9966
0.33	−4.50	1,999	−230,260	0.9823	−6.23	3,017	−369,127	0.9889

## Results and Discussion

Organic solvents exhibit weak adsorption in chromatographic system in comparison with most of the organic compounds. Solvents are usually eluted in the void volume of the chromatographic column. However, at high water concentration in the binary mobile phase, the significant retention of organic solvent may be found.

**Fig. 1:**
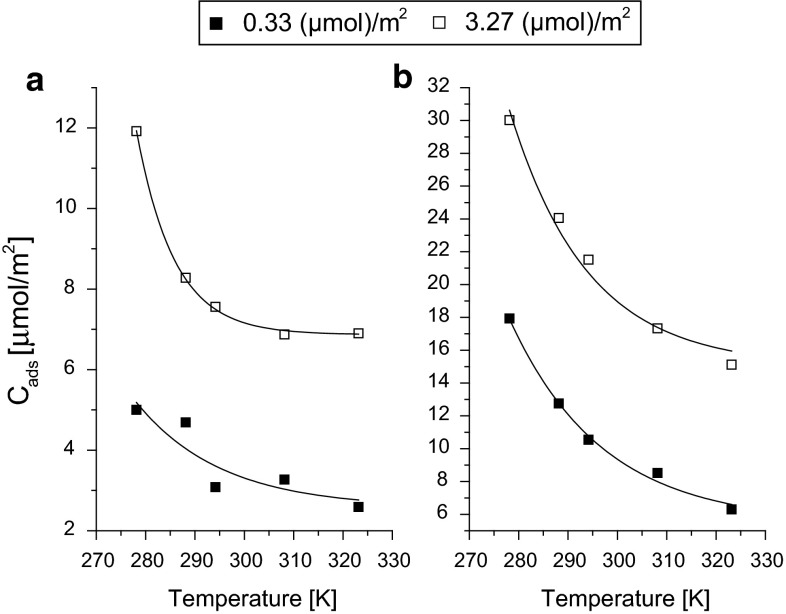
Influence of the temperature on the maximum concentration of the adsorbed solvent (*C*_ads_): methanol (**a**) and acetonitrile (**b**). Data according to results presented in [34]

The temperature dependences were investigated on five packed octadecyl columns with different coverage densities (from 0.33 up to 3.27 μmol m^−2^). As seen in Fig. 1, adsorption of both solvents decreases with the temperature in non-linear manner.

In the case of methanol (Fig. 1a), the highest decrease was observed on high coverage density phase (*α*_RP_ = 3.27 μmol m^−2^) in the range 278–288 K. This decrease was about 30 % of the initial value. For low coverage density phase, the decrease of the adsorbed methanol with increase of the temperature was much smaller. The relative decrease of methanol adsorption in the whole range of tested temperatures 278–323 K was 55 and 40 % for low coverage phase (0.33) and high coverage phase (3.27 μmol m^−2^). It is known that the adsorption of methanol is much weaker than adsorption of acetonitrile [31, 34]. Thus, it can be expected that the temperature influences acetonitrile adsorption much stronger (Fig. 1b). The relative decrease of acetonitrile adsorption in the whole range of tested temperatures 278–323 K was 70 and 45 % for low coverage phase (0.33 μmol m^−2^) and high coverage phase (3.27 μmol m^−2^), respectively. It may be concluded that considerable influence of temperature on adsorption is observed when the modifier strongly solvates the stationary phase. The decrease of solvent adsorption will result in the decreasing of the elution strength. Thus, the retention of given solute does not changes tremendously with the temperature.

**Fig. 2:**
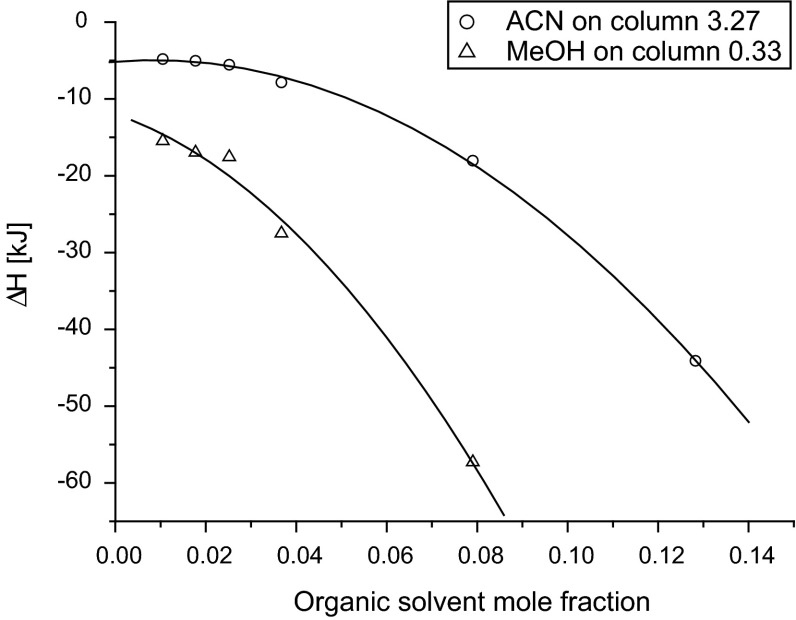
Changes of the enthalpy of MeOH and ACN with mole fraction of organic solvent in the mobile phase at 298.15 K

Adsorption of methanol from water solution exhibits higher enthalpy than in the case of acetonitrile, which may be attributed to possibility of hydrogen bond creation, which was proven in the previous study [35]. In Fig. 2, the changes of methanol and acetonitrile enthalpies with the organic solvent mole fraction in the mobile phase at the temperature 298.15 K are presented. For both solvent, the adsorption process is exothermal. The enthalpy increases (gives more negative values) when the concentration of organic solvent in the mobile phase increases. It is a result of competitive adsorption of water and organic modifiers on the polar residual silanols at stationary-phase surface. When water molecules block most of the polar adsorption sites, the enthalpy of solvent adsorption decreases because organic solvent molecules adsorb mostly on bonded ligands due to weak dispersive forces. Similar results are presented in [36], where water–methanol mixtures provide lower thermal effect (enthalpy) of stationary-phase immersion than pure methanol.

**Fig. 3:**
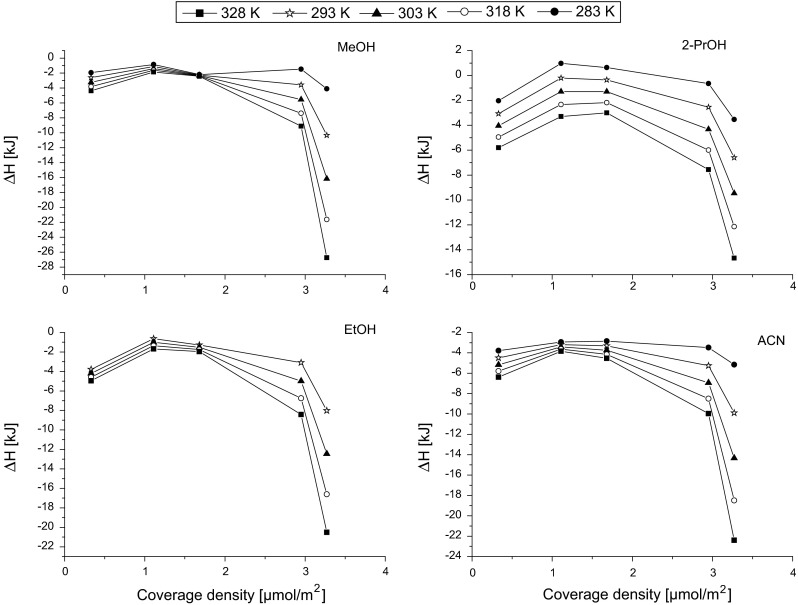
Changes of the enthalpy of MeOH, 2-PrOH, EtOH and ACN adsorption with surface coverage density of octadecyl stationary phases measured at 95 % of water in the mobile phase

Changes of the enthalpy and entropy of solvent adsorption were measured using mobile phase containing 95 % of water in the mobile phase. For a series of octadecyl-bonded phases with different coverage densities in the range from 0.33 up to 3.27 μmol m^−2^, the enthalpy of all solvents changes in parabolic manner. The lowest values were obtained for stationary phase with *α*_RP_ = 1.11 μmolm^−2^ for all solvents (see Fig. 3). Enthalpies of all solvents on the stationary phase with the lowest surface coverage (and the highest concentration of residual silanols) were slightly higher. Next, the significant increase of the enthalpies was observed, when coverage density of packing materials was extremely high (3.27 μmol m^−2^). The biggest differences are observed between stationary phases 2.95 and 3.27 μmol m^−2^. The reason of such phenomenon may be the change of the interaction mechanism. This stationary phase has extreme coverage density and the migration of solvent molecules between them is hampered. In addition, the competitive adsorption of water is reduced, thus the adsorption of organic solvent is stronger.

Among the four solvents, the highest values of enthalpy (−Δ*H*) were observed for methanol—the smallest and the most polar molecule. The lowest enthalpy was measured for propan-2-ol which molecule is the biggest and the most hydrophobic from the tested group. It suggests that solvent enthalpy depends on the polar interaction with residual silanols and on the possibility of penetration between the bonded ligands of the stationary phase that depends on the size of the molecule.

Enthalpies of adsorption from water of all solvents increase with the temperature increase. Also the changes of the solvent enthalpy with surface coverage are more significant at higher temperature. Changes of the enthalpies with temperatures confirm that temperature influences more strongly the solvation of high coverage density phases that is shown in Fig. 1.

Another interesting phenomenon is the similarity of the enthalpies for the stationary phase with coverage density 1.65 μmol m^−2^ at different temperatures, for methanol and ethanol. For this material, the enthalpy is almost independent of the temperature. In the case of acetonitrile, such point is shifted to the material with lower surface coverage (1.11 μmol m^−2^). This situation was not observed for propan-2-ol.

Parabolic trend of the enthalpy changes may be a result of two different phenomena. First, there are the polar interactions with residual silanols, when the coverage of the bonded ligands is low. When the concentration of bonded ligands increases, the number of residual silanols decreases so the enthalpy decreases as well. However, further increase of the surface coverage density causes the formation of dense bonded layer. Thus, the most dominant process is the solvation of huge number of octadecyl ligands via dispersive interactions. As a result, the enthalpy increases with the increased number of bonded ligands on the stationary-phase surface.

**Fig. 4:**
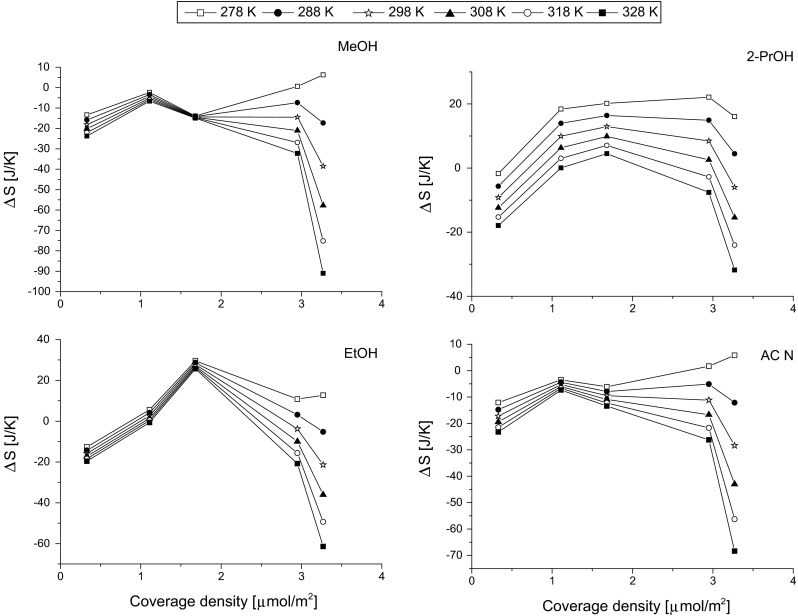
Changes of the entropy of the MeOH, 2-PrOH, EtOH and ACN adsorption from water with surface coverage density of octadecyl stationary phases

Similar parabolic trend is observed for entropy. The changes of the entropy for all tested solvents in the temperature range from 278 to 328 K are shown in Fig. 4. In the case of MeOH and ACN, the lowest values were obtained for stationary phase with coverage density 1.11 μmol m^−2^. However, at 278 K the minimum is shifted to the highest covered phases. Entropy on low coverage density phases is almost independent of the temperature for MeOH, EtOH and ACN, maximum 10 J K^−1^ in the temperature range from 278 to 328 K. The entropy changes in the case of propan-2-ol are most significant. On the other hand, temperature influences strongly the entropy of MeOH, EtOH and ACN on high covered phases.

It has to be mentioned that entropy for MeOH and ACN reaches mostly negative values whereas for EtOH and 2-PrOH the positive values are observed. In the case of the enthalpy changes, which was shown in Fig. 3, the positive values were observed only for propan-2-ol. From the tested solvent, ethanol and propan-2-ol have the highest hydrophobicity (the longest alkyl chain). A positive Δ*S* value indicates a decrease in order of the chromatographic system as the solvent molecule is transferred from the mobile phase to the stationary phase, which is the evidence of the hydrophobic effect [11].

In the case of Gibbs free energy (Δ*G*) listed in Table 4, the negative values are observed for ACN, whereas MeOH provides positive values. The negative values of ACN Δ*G* confirm the adsorption process on the stationary phase. The positive values of Δ*G* in the case of MeOH are against adsorption process. However, it has to be remembered that experiments were carried out at high water content in the mobile phase. As it was proven in the previous study [37], adsorption of MeOH and water is a competitive phenomenon. Adsorption of MeOH is also much weaker in the comparison with ACN. It has to be mentioned that for the lowest covered stationary phase (0.33 μmol m^−2^), the Δ*G* of ACN is also positive that may confirm the theory of significant adsorption of water on the residual silanols and the displacement effect of organic modifier by adsorption of water.

**Table 4:** Enthalpies, entropies and Gibbs free energy of methanol and acetonitrile calculated from curvilinear van’t Hoff model Eqs. (4, 5)

*α*_RP_^*I*^ (μmol m^−2^)	−Δ*H*°^278.15^	Δ*S*°^278.15^	Δ*G*° ^278.15^	−Δ*H*°^288.15^	Δ*S*°^288.15^	Δ*G*°^288.15^	−Δ*H*°^298.15^	Δ*S*° ^298.15^	Δ*G* ^o298.15^	−Δ*H*°^308.15^	Δ*S*° ^308.15^	Δ*G*°^308.15^	−Δ*H*°^318.15^	Δ*S*°^318.15^	Δ*G*°^318.15^	−Δ*H* ^° 328.15^	Δ*S* ^° 328.15^	Δ*G* ^° 328.15^
ACN																		
3.27	0.06	5.83	−1.70	5.15	−12.15	−1.60	9.90	−28.35	−1.40	14.34	−42.99	−1.11	18.50	−56.28	−0.66	22.40	−68.37	0.07
2.95	1.57	1.67	−2.04	3.48	−5.08	−2.00	5.27	−11.17	−1.94	6.93	−16.67	−1.80	8.50	−21.66	−1.61	9.96	−26.20	−1.35
1.68	2.34	−6.14	−1.66	2.84	−7.90	−1.61	3.31	−9.49	−1.57	3.74	−10.93	−1.50	4.15	−12.24	−1.45	4.54	−13.43	−1.32
1.11	2.67	−3.48	−1.28	2.94	−4.41	−1.18	3.18	−5.25	−1.14	3.41	−6.01	−1.04	3.63	−6.70	−0.89	3.83	−7.33	−0.83
0.33	3.02	−12.11	0.36	3.78	−14.81	0.47	4.50	−17.25	0.60	5.16	−19.46	0.86	5.79	−21.46	1.07	6.38	−23.28	1.23
MeOH																		
3.27	−2.55	6.24	0.79	4.12	−17.32	0.90	10.34	−38.56	1.21	16.16	−57.76	1.59	21.62	−75.18	2.20	26.74	−91.03	3.19
2.95	−0.77	0.61	0.59	1.49	−7.35	0.64	3.59	−14.53	0.75	5.56	−21.02	0.09	7.40	−26.90	1.16	9.13	−32.25	1.43
1.68	2.14	−13.91	0.69	2.21	−14.16	0.85	2.27	−14.38	0.93	2.34	−14.59	0.97	2.39	−14.77	1.14	2.45	−14.94	1.26
1.11	0.56	−2.32	0.85	0.86	−3.37	0.96	1.13	−4.31	1.00	1.39	−5.17	1.09	1.63	−5.94	1.19	1.86	−6.65	1.34
0.33	1.25	−13.34	2.46	1.96	−15.87	2.64	2.63	−18.14	2.75	3.25	−20.20	2.95	3.83	−22.06	3.19	4.38	−23.76	3.41

Comparison of the entropy changes presented in Fig. 4 reveals that the entropy driving solvent molecule into the low coverage density stationary phase is greater than the entropy driving benzene into the high coverage stationary phase. It may indicate a relatively stronger entropic expulsion from the higher density phase, which is consistent with the interphase theory of retention [11] and it was observed in the previous study [38]. The penetration of solvent molecules through a dense film of bonded ligands is hampered in the comparison with low surface coverage phase.

Changes of the enthalpy and entropy over the coverage density of octadecyl phases exhibit similar trend to variation of organic solvent adsorbed amount. As it was discussed in the previous study, as the surface coverage increases, the excess amount of adsorbed solvent increases as well until the effect of the decreasing surface area caused by surface modification becomes significant. Then, due to hampered penetration through the bonded ligands, the amount of adsorbed solvent decreases. Solvent molecules adsorb only on the top of the ligand, however, further increase of surface coverage causes that the portion of solvent molecules is distributed between mobile phase and octadecyl-bonded layer due to partition mechanism [31].

**Fig. 5:**
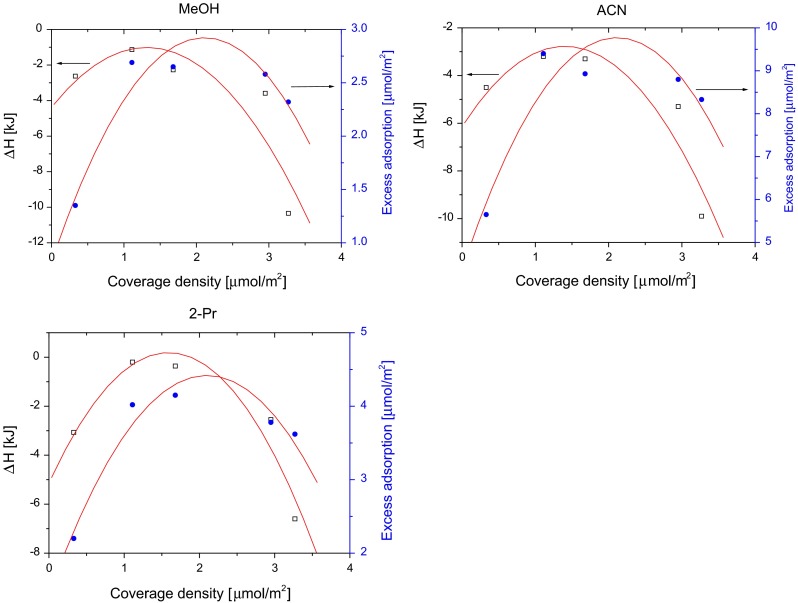
Changes of the enthalpy and maximum excess adsorbed amount of solvent (maximum of the excess adsorption isotherm measured at 303 K) with the coverage density of octadecyl-bonded phases

The similarity of enthalpy and adsorption values is compared in Fig. 5. As was discussed above, the higher values of enthalpy are observed, when the adsorption of given solvent increases. For the stationary phases with medium surface coverage, the lowest values of both parameters are observed.

## Conclusions

Temperature is important parameter which influences the retention and solvation in liquid chromatography system. The increase of the temperature causes the decrease of the retention and adsorption of organic solvent used as a organic modifier of the mobile phase. Changes of the enthalpy are related with the variation of amount of adsorbed solvent. The highest values are observed for extreme low and high coverage density phases and the minimum values were measured for stationary phase with medium surface coverage. Entropy of solvation process changes in similar manner to enthalpy.

Both the enthalpy and entropy increase at higher temperature. The changes of entropy and enthalpy with temperature increase are the highest for high coverage density phases. Once again, the enthalpy and entropy on stationary phases with medium surface coverage are almost independent on the temperature. Thus, it seems that temperature has weak influence on the solvation and retention on medium covered phase in the comparison with high density phases, where temperature has important influence on both, solute retention and solvation process.
